# Editorial: Pulmonary Innate Lymphoid Cells - Gatekeepers of Respiratory Health

**DOI:** 10.3389/fimmu.2022.871207

**Published:** 2022-03-01

**Authors:** Malcolm R. Starkey, Hitesh Deshmukh, Nicholas W. Lukacs, Clare M. Lloyd

**Affiliations:** ^1^ Department of Immunology and Pathology, Central Clinical School, Alfred Research Alliance Monash University, Melbourne, VIC, Australia; ^2^ Division of Neonatology, Cincinnati Children’s Hospital Medical Center, Cincinnati, OH, United States; ^3^ Division of Pulmonary Biology, Cincinnati Children’s Hospital Medical Center, Cincinnati, OH, United States; ^4^ Center for Inflammation and Tolerance, Cincinnati Children’s Hospital Medical Center, Cincinnati, OH, United States; ^5^ Department of Pediatrics, University of Cincinnati College of Medicine, Cincinnati, OH, United States; ^6^ Department of Pathology, University of Michigan, Ann Arbor, MI, United States; ^7^ Mary H. Weiser Food Allergy Center, University of Michigan, Ann Arbor, MI, United States; ^8^ National Heart and Lung Institute, Imperial College London, London, United Kingdom

**Keywords:** innate lymphoid cells, ILC, lung, airway, respiratory, pulmonary, infection, disease

The Research Topic “*Pulmonary Innate Lymphoid – Gatekeepers of Respiratory Health*”, provides a series of up-to-date reviews including a fresh look at innate lymphoid cell (ILC) development (Shin et al.); the fate of activated ILC2 (Wirtz et al.); the role of ILC2 and ILC3 in pulmonary infections (Fonseca et al. and Hoffmann et al.) and an update on ILCs in chronic respiratory diseases (Hsu et al. and Rao et al.). These reviews provide the ideal framework to enable this editorial to focus on the six original research articles in the collection that bring new knowledge to the field of pulmonary ILC biology.

## c-Rel Is Required for ILC2 Activation and Pulmonary Inflammation

ILC2 activation is governed by a network of transcriptional regulators including nuclear factor (NF)-kB family transcription factors. While it is known that activating interleukin (IL)-33 receptor signaling results in downstream NF-kB activation, the underlying molecular mechanisms remain elusive (Mindt et al.).

In this Research Topic, back-to-back papers demonstrate that the NF-kB subunit c-Rel is required to mount effective pulmonary type 2 immune responses (Mindt et al. and Zaini et al.). IL-33-mediated activation of ILC2 *in vitro* as well as *in vivo* induced c-Rel mRNA expression and increased c-Rel protein levels (Mindt et al.). Furthermore, IL-33-mediated activation of pulmonary ILC2 caused nuclear translocation of c-Rel (Mindt et al.). Although c-Rel was found to be a critical mediator of pulmonary type 2 immune responses, ILC2-intrinsic deficiency of c-Rel did not influence the developmental capacity of ILC2 nor affected homeostatic numbers of lung-resident ILC2 at steady state (Mindt et al. and Zaini et al.). Moreover, ILC2-intrinsic deficiency of c-Rel alters the capacity of ILC2 to upregulate the expression of the key stimulatory receptors ICOSL and OX40L, and the expression of the signature type 2 cytokines IL-5, IL-9, IL-13, and granulocyte-macrophage colony-stimulating factor (Mindt et al.). c-Rel-deficient mice displayed significantly reduced lung inflammation in response to pulmonary challenge with either papain or recombinant IL-33 (Mindt et al. and Zaini et al.).

Collectively, c-Rel promotes ILC2-driven allergic airway inflammation and suggest that c-Rel may contribute to the pathophysiology of ILC2-mediated allergic airway disease. c-Rel thereby represents a promising future target for the treatment of allergic asthma. (Mindt et al. and Zaini et al.). However, the role of c-Rel in allergen-induced asthma models e.g., following instillation of house dust mite and the impact on functional parameters such as airway hyperresponsiveness and mucus secreting cell metaplasia, remain to be elucidated.

## Putting the STING into ILC2-ILC1 Shifting With cyclic-di-GMP

Type 2 inflammation underpins several endotypes of asthma. In asthma, recurrent viral infections, bacterial colonization, and host cell death drive the accumulation of intracellular cyclic-di-nucleotides including cyclic-di-GMP (CDG). However, the impact of CDG on allergic airway inflammation is unknown. To explore this, Cavagnero et al. intranasally administered CDG, which induced early airway type 1 interferon (IFN) production and suppressed IL-7R^+^ ST2^+^ ILC2 and type 2 lung inflammation, following pulmonary challenge with either Alternaria or recombinant IL-33 (Cavagnero et al.). An IL-7R^–^ST2^–^CD90.2^+^ lung ILC subset, that had a transcriptional signature that was consistent with ILC1, were expanded by administration of CDG when it was delivered in combination with either the fungal allergen, Alternaria, or recombinant IL-33. CDG-mediated suppression of pulmonary type 2 inflammation occurred independently of IL-18R, IL-12, and STAT6, but required the stimulator of interferon genes (STING) and type 1 IFN signaling (Cavagnero et al.).

Collectively, this study demonstrates that CDG drives STING-dependent IFN production, ILC1 activation and accumulation, as well as ILC2 suppression and abrogation of innate type 2 innate airway inflammation. This study adds to our understanding of how the pathogenesis of allergic airway disease may be impacted by lung insults due to cellular stress, bacterial and viral infections (Cavagnero et al.).

## The α7nAChR Agonist PNU-282987 Inhibits ILC2 Function and Allergic Airway Inflammation

The cholinergic anti-inflammatory pathway controls inflammation through the release of the neurotransmitter acetylcholine. Acetylcholine can also stimulate the a7 nicotinic acetylcholine receptor (a7nAChR) that is highly expressed on ILC2. The a7nAChR agonist, GTS-21, is known to attenuate ILC2-dependent airway hyperreactivity in mice. In this Research Topic, Yuan et al. explore the ability of an alternate a7nAChR agonist, PNU-282987, to suppress ILC2-mediated allergic airway inflammation (Yuan et al.). Both PNU-282987 and GTS-21 significantly reduced airway mucus secreting cell hyperplasia, eosinophil infiltration into the airways, and ILC2 numbers in bronchoalveolar lavage fluid, following respiratory challenge with recombinant IL-33 or Alternaria (Yuan et al.). In summary, PNU-282987 inhibited ILC2-associated airway inflammation, comparable to that of GTS-21.

## Rhinovirus C Infection Induces ILC2 Expansion and Airway Inflammation

Rhinovirus C (RV-C) infection is associated with severe asthma exacerbations. Rajput et al. hypothesized that RV-C infection, in contrast to RV-A, would preferentially stimulate type 2 inflammation, leading to exacerbated eosinophilic airway inflammation. To test this hypothesis the team developed a novel mouse model of RVC infection. Mice inoculated with RV-C15 showed lung viral titers of 1 x 10^5^ TCID50 units 24 h after infection, with levels declining thereafter (Rajput et al.). IFN-α, β, γ and λ2 mRNA expression peaked 24-72 hours post-infection. Compared to RV-A1B, mice infected with RV-C15 demonstrated higher bronchoalveolar eosinophils, mRNA expression of IL-5, IL-13, IL-25, Muc5ac and Gob5, protein production of IL-5, IL-13, IL-25, IL-33 and TSLP, and expansion of ILC2 (Rajput et al.). In contrast to ILC2-sufficient *Rora* ^fl/fl^ littermates, RV-C-infected ILC2-deficient *Rora* ^fl/fl^ *Il7r* ^cre^ mice failed to show eosinophilic inflammation or mRNA expression of *Il13*, *muc5ac* and *muc5b*. It was concluded that, compared to RV-A1B, RV-C15 infection induces ILC2-dependent type 2 airway inflammation, providing new insights into the mechanism of RV-C-induced asthma exacerbations (Rajput et al.).

## Children With Asthma Have Increased Circulating ILC2 and NCR^-^ ILC3

Asthma is the most frequent cause of hospitalization among children; however, little is known regarding the effects of asthma on immune responses in children. Hosseini et al. aimed to evaluate peripheral blood mononuclear cell composition in children with and without asthma. They found that the frequency of circulating ILC2 and NCR^-^ ILC3 were significantly higher in asthmatics compared to non-asthmatic controls. There was no change in the frequency of other leukocyte subsets commonly associated with asthma such as eosinophils and CD4^+^ T helper cells.

## Conclusion

The collection of original articles and reviews presented in this Research Topic highlight the ability of pulmonary ILC to modulate the severity of allergic airway inflammation, respiratory infections, and respiratory diseases. Specific advances were made in describing the role for the NF-kB subunit c-Rel in activation and effector function of pulmonary ILC2 in mice; showing that CDG induces a ILC2-ILC1 shift that may reduce type 2 inflammation and promote antimicrobial ILC1 responses *in vivo*; demonstrating that the a7nAChR agonist PNU-282987 attenuates ILC2-associated airway inflammation; evidence that respiratory RV-C infection induces ILC2 and eosinophilic airway inflammation in mice; and that children with asthma have increased circulating ILC2 and NCR^-^ ILC3.

## Author Contributions

MS led the research collection and wrote the editorial. HD, NL, and LL edited manuscripts for the Research Topic and the editorial. All authors contributed to the article and approved the submitted version.

## Conflict of Interest

The authors declare that the research was conducted in the absence of any commercial or financial relationships that could be construed as a potential conflict of interest.

## Publisher’s Note

All claims expressed in this article are solely those of the authors and do not necessarily represent those of their affiliated organizations, or those of the publisher, the editors and the reviewers. Any product that may be evaluated in this article, or claim that may be made by its manufacturer, is not guaranteed or endorsed by the publisher.

